# Entropy-Based Statistics and Their Applications

**DOI:** 10.3390/e25060936

**Published:** 2023-06-14

**Authors:** Zhiyi Zhang

**Affiliations:** Department of Mathematics and Statistics, UNC Charlotte, Charlotte, NC 28223, USA; zzhang@charlotte.edu

During the last few decades, research activity in modeling the properties of random systems via entropies has grown noticeably across a wide spectrum of fields. From the early days of statistical thermodynamics, the concept of entropy has evolved into many practically useful tools. For example, in modern data science, many powerful methodologies in artificial intelligence and machine learning are developed with logic arguments firmly anchored in entropies and their statistical estimation from sample data. As in any statistical exercise, the issue of reliability of estimated entropies and the derived inferences becomes increasingly acute and hence is in need of careful consideration within a clear statistical framework.

This Special Issue, under the theme of “Entropy-Based Statistics and its Applications”, which may be more concisely termed “Entropic Statistics”, aims to collect unpublished research contributions on this topic in theory and in application. This Special Issue is intended to serve as a forum for the presentation of and the discourse on novel ideas and approaches in entropic statistics.

In a broader perspective, the increased activity in statistical exercises via entropies may suggest a shift in focus of the foundation of statistical science, away from a richly metrizable real space where random variables reside toward a non-metrized and non-ordinal countable alphabet, where more general random elements reside. In a non-metrized sample space, many familiar notions usually associated with random variables are no longer available, including, for example, moments, neighborhoods, cumulative distribution functions, and tails. Without these useful notions and the rich theory behind them, the mathematical tools for statistics associated with random elements on alphabets are greatly depleted in comparison to the traditional statistics associated with random variables. Entropies could indeed provide a means to replenish this depleted tool-box to support fuller mathematical maneuvers for the purpose of statistical inference on alphabets.

Many fundamental questions may be considered fruitfully in a more holistic framework of entropic statistics. For example, what is entropy? To many, if not most, entropy is a particular function of the probability distribution, $p=\{p_{k};k\geq 1\}$, on a countable alphabet, $X=\{\ell_{k};k\geq 1\}$, of the form $H_{BGS}\left(p\right)=-\sum_{k\geq 1}p_{k}\ln p_{k}$, often referred to as the Boltzmann–Gibbs–Shannon entropy. The Boltzmann–Gibbs–Shannon entropy has deep roots and uses in thermodynamics. However, in a holistic framework, it is merely a single-value index describing a profile state of the underlying random system. Many entropy indices have been defined and studied in existing literature and across diverse fields, including physics and information theory. Many indices also come under the banner of diversity indices. While serving different interests, the most noticeable common property shared by these entropies is that of being label-independent (with respect to the letters of the alphabet) functions of the underlying distribution, p. Could the property of label independence serve as a basis for a more general definition of entropy? If a class of general entropies could be defined, then the central objective of making inferences about the underlying distribution may be served more effectively without obligatory references to some named entropies such as the Boltzmann–Gibbs–Shannon entropy.

If entropic statistics is thought of as a collection of inferential statistical methodologies describing the stochastic nature of the underlying random system based exclusively on entropies, then one would want to understand what types of properties are describable and to what extent. What are the advantages of subscribing to a framework of entropic statistics? What are the limitations? More concretely, in estimating an entropy, it would be of interest to understand what modes of performance measures are appropriate and what types of convergence theorems may be established. Discussions on these topics would add great value to this Special Issue.

If, as it is often said, “necessity is the mother of invention”, a sound theory should be born of good applications. Reports of applied studies, based on the estimation of well-justified entropies and rigorously gauged statistical reliability, are not only welcome but absolutely vital to the theme of this Special Issue. 

